# Mediterranean Diet and Cardiovascular Disease: A Critical Evaluation of *A Priori* Dietary Indexes

**DOI:** 10.3390/nu7095367

**Published:** 2015-09-16

**Authors:** Annunziata D’Alessandro, Giovanni De Pergola

**Affiliations:** 1 Endocrinologist, General Practitioner, General Medicine ASL BA/4 D.S.S. 8, viale Japigia 38/G, Bari 70126, Italy; 2 Department of Biomedical Sciences and Human Oncology, Section of Internal Medicine and Oncology, School of Medicine, Policlinico, Piazza Giulio Cesare 11, University of Bari “Aldo Moro”, Bari 70124, Italy; E-Mail: gdepergola@libero.it

**Keywords:** Mediterranean diet, cardiovascular disease, coronary heart disease, cerebrovascular disease, *a priori* dietary indexes

## Abstract

The aim of this paper is to analyze the *a priori* dietary indexes used in the studies that have evaluated the role of the Mediterranean Diet in influencing the risk of developing cardiovascular disease. All the studies show that this dietary pattern protects against cardiovascular disease, but studies show quite different effects on specific conditions such as coronary heart disease or cerebrovascular disease. *A priori* dietary indexes used to measure dietary exposure imply quantitative and/or qualitative divergences from the traditional Mediterranean Diet of the early 1960s, and, therefore, it is very difficult to compare the results of different studies. Based on real cultural heritage and traditions, we believe that the *a priori* indexes used to evaluate adherence to the Mediterranean Diet should consider classifying whole grains and refined grains, olive oil and monounsaturated fats, and wine and alcohol differently.

## 1. Introduction

Diet is a very complex exposure variable in nutritional epidemiology. In the last years, the attention of scientists has moved from single foods or nutrients to dietary patterns, namely, the overall food consumption in the diet, that is, a holistic approach to study items that are highly interrelated and influence their impact on the risk of non-communicable chronic diseases [1]. There are two distinct approaches to examine overall dietary exposure: *a priori* and *a posteriori*. The first is based on the construction of an index on the basis of knowledge about the relationship between foods and diseases (sometimes synthesized in dietary guidelines) [2] or food consumption models (Mediterranean type diet, prudent diet, step I American Heart Association diet, and so on) [3]. The index is then used as a dietary exposure variable in epidemiological studies [2]. Conversely, the *a posteriori* approach is based on multivariate statistical techniques that identify sets of dietary exposures strongly correlated together and not previously known [3].

The *a priori* approach has been widely used in epidemiological studies to evaluate the relationship between the Mediterranean Diet and several outcomes such as cardiovascular incidence and mortality, risk factors for cardiovascular diseases, and incidence of major chronic non-communicable diseases.

We present a description of *a priori* dietary indexes used in the epidemiological studies that examined the relationship between the Mediterranean Diet and cardiovascular disease in this article. Then, we proceed to critically evaluate them.

## 2. Methods

PubMed was searched until May 2015 to find publications on the Mediterranean Diet and cardiovascular disease, using “Mediterranean Diet” matched with “coronary heart disease”, “cerebrovascular disease”, “stroke”, “cardiovascular disease”, “dietary score” and “dietary index” as key words.

## 3. Results

We found 26 studies that evaluated the relationship between the Mediterranean Diet and cardiovascular disease (Table 1). We also found five original *a priori* dietary indexes used to evaluate dietary exposure in such studies: *Mediterranean Diet Score* [4], *Dietary Score* [5], *Mediterranean Adequacy Index* [6], *a priori Mediterranean Dietary Pattern* [7], *PREvención con DIeta MEDiterránea* (PREDIMED) score [8]. Moreover, we found nine indexes derived from the original Mediterranean Diet Score (Table 2). We found only one index, the Mediterranean-Style Dietary Pattern Score, used to assess the conformity of an individual’s diet to a traditional Mediterranean Diet [9].

**Table 1:** *A priori* dietary indexes and main results of the studies that evaluated the relationship between the Mediterranean Diet and CVD, CHD and cerebrovascular disease.

Authors (Year) (Reference)	*A priori* Index	Subjects, Number, Age	Study’s Name, Type of Study and Follow-Up	Main Results
Bilenko *et al.*, (2005) [10] °	MDS	Israeli Jewish population, 1159 adults, ≥35 year	NNS, transverse study	In men for each MDS decrease significant increased risk for MI, CABG, PTCA, CVD. In women similar trend, but not statistical significance.
Hoşcan *et al*., (2015) [11] °	MDS	Turkish population, 900 adults, 25–70 year	Cohort, (5.1 year)	Men with a lower adherence to the Mediterranean Diet had a significantly higher risk of CHD morbidity compared to men with a higher adherence. No association was found in women.
Trichopoulou *et al*., (2003) [12]	t-MED	Greek population, 22,043 adults, 20–86 year	EPIC, cohort, (3.7 year)	A 2-point increase in t-MED is associated with a CHD mortality reduction by 33%.
Dilis *et al*., (2012) [13]	t-MED	Greek population, 23,572 adults, 20–86 year	EPIC, cohort, (10 year)	A 2-point increase in t-MED was associated with a decrease in CHD mortality by 22% (*p* = 0.003) and a non-significant reduction in CHD incidence
Misirli *et al*., (2012) [14]	t-MED	Greek population, 23,601 adults 20–86 year	EPIC, cohort, (10.6 year)	A 2-point increase in t-MED was associated with a significant decrease in cerebrovascular disease incidence and a non-significant decrease in cerebrovascular disease mortality
Tsivgoulis *et al*., (2015) * [15]	t-MED	U.S. population, 20,197 adults, 65 ± 9 year	REGARDS, cohort, (6.5 year)	A 1-point increase in t-MED was independently associated with a 5% reduction in the risk of incident ischemic stroke. No association with incident hemorrhagic stroke
Martínez-González *et al*., (2011) [16]	t-MED	Spanish population, 13,609 young, mean 38 year	SUN, cohort, (4.9 year)	A 2-point increase in t-MED was associated with a 20% decrease in total CVD risk and to a 26% reduction in CHD risk
Gardener *et al*., (2011) * [17]	t-MED	U.S. population, 2568 adult, mean 69 ± 10 year	NOMAS, cohort (9 year)	A 1-point increase in t-MED was associated with a 9% (*p* < 0.05) decrease in risk of vascular death. No association was found for vascular events (ischemic stroke and MI)
Agnoli *et al*., (2011) [18]	t-MED	Italian population, 40,681 adults, 35–74 year	EPICOR, cohort , (7.89 year)	t-MED was inversely associated with the risk of ischemic stroke and positively with the risk of hemorrhagic stroke without statistical significance
Turati *et al*., (2015) [19]	t-MED	Italian population, 760 patients with a first episode of non-fatal MI/682 controls, 16–79 year	Case-control	A 1-point increase in the t-MED was associated with a reduced risk of a first episode of MI by 9%
Knoops *et al*., (2004) [20]	The score according to Knoops	European population (11 European countries), 2339 elderly people, 70–90 year	HALE study, cohort, (10 year)	A score of at least four points reduced the CHD mortality by 39% and the CVD mortality by 29%
Agnoli *et al*., (2011) [18]	The Italian Mediterranean Index	Italian population, 40,681 adults, 35–74 year	EPICOR, cohort, (7.89 year)	The Italian Mediterranean Index was inversely associated with ischemic stroke (*p* for trend = 0.001) and hemorrhagic stroke (*p* for trend = 0.07)
Fung *et al*., (2009) [21]	a-MED	U.S. population, 74,886 females nurses, 38–63 year	NHS, cohort, (20 year)	Women in the highest a-MED quintile were at lower risk for both total CHD and total stroke compared with those in the lowest quintile (*p* for trend = 0.001 and = 0.03, respectively)
Mitrou *et al*., (2007) [22]	a-MED	U.S. population, 380,296 adults, median age 62 year	NIH-AARP Diet and Health Study, cohort (5 year)	The risk of mortality for CVD was lower in men and women with higher adherence to the Mediterranean Diet compared to those with a lower adherence (*p* < 0.001 and *p* = 0.01, respectively)
Buckland *et al*., (2009) [23]	r-MED	Spanish population, 41,078 adults, 29–69 year	EPIC, cohort, (10.4 year)	A 1-point increase in the r-MED was associated with a 6% lower risk of total CHD (*p* for trend < 0.001)
Hoevenaar-Blom *et al*., (2012) [24]	m-MED	Dutch population, 34,708 adults, 20–70 year	EPIC, cohort, (10–15 year)	A 2-point increase in the m-MED was inversely and significantly associated with fatal CVD, composite CVD, incident MI, incident stroke, and pulmonary embolism
Sjögren *et al*., (2010) [25]	The score according to Sjogren	Swedish men, 924 elderly, 71 ± 1 year	Cohort, (10.2 year)	A higher adherence to the Mediterranean Diet was associated with a lower risk of CVD mortality as compared to lower adherence (*p* for trend = 0.009)
Tognon *et al*., (2012) [26]	The score according to Tognon 2012	Sweden population, 77,151 adults, 30–70 year	VIP, cohort , (median 9 year)	The score according to Tognon 2012 was significantly associated only in women but not in men with mortality for CVD and mortality for MI. No association was found with stroke mortality in both genders.
Tognon *et al*., (2014) [27]	The score according to Tognon 2014	Danish population, 1849 adults	MONICA project, longitudinally	The score according to Tognon 2014 was inversely associated with CVD incidence and mortality. The strength of the associations depended on the way in which the score was built (see text)
Panagiotakos *et al*., (2015) [28]	DS	Greek population, 2583 adults, 18–89 year	ATTICA study, cohort, (10 year)	A 1-point increase in the DS decreased CVD risk by 4%
Panagiotakos *et al*., (2015) [29]	DS	Greek population, 848 patients with a first symptom of CHD/1078 controls	CARDIO 2000, case-control	An 11/55 unit increase in DS was associated with a reduced odds of having a first acute coronary syndrome by 27%
Kastorini *et al*., (2011) [30]	DS	Greek population, 250 patients with a first episode of acute coronary syndrome and 250 patients with a first ischemic stroke/500 controls	Case-control	A 1-point increase in the DS reduced the odds of having acute coronary syndrome by 9% and of having a stroke by 12%
Kastorini *et al*., (2012) [31]	DS	Greek population, 250 patients with a first ischemic stroke/250 controls	Case-control	A 1-point increase in DS reduced the odds of having a first ischemic stroke by 17% in non-hypercolesterolemic participants and by 10% in hypercolesterolemic participants
Fidanza *et al*., (2004) [32]	MAI	USA, Europe, Japan, 12,763 men, 40–59 year	Seven Countries Study, cohort, (25 year)	The MAI was inversely correlated with death rates from CHD (*p* = 0.001) in 16 cohorts of Seven Countries Study
Menotti *et al*., (2012) [33]	MAI	Italian population, 1139 men, 45–64 year	Seven Countries Study, two Italian cohorts, (20–40 year)	The hazard ratio for 2.7 units of MAI was associated with a CHD mortality reduction of 26% in 20y and 21% in 40y of follow-up
Martínez-González *et al*., (2002) [7]	*a priori* Mediterranean Dietary Pattern	Spanish population, 171 patients with a first MI/171 controls, <80 year	Case-control	A 1-point increase in the *a priori* Mediterranean Dietary Pattern was associated with a reduced risk of 8% for a first MI (*p* < 0.01)
Estruch *et al*., (2013) [34]	PREDIMED score	Spanish population, 7447 adults, 50–80 year	Randomized trial (4.8 year)	The rate of major CVD events was reduced by 30% (*p* = 0.01) in the group assigned to the Mediterranean Diet with extra-virgin olive oil and by 28% (*p* = 0.03) in the group assigned to the Mediterranean Diet with nuts compared with the control group. In subgroup analyses the supplemented Mediterranean Diet was significantly protective towards stroke but not towards MI and CVD deaths in comparison with the control diet

MDS, Mediterranean Diet Score; t-MED, Trichoupoulou Mediterranean Diet Index; a-MED, alternate Mediterranean Diet Index; r-MED, relative Mediterranean Diet Index; m-MED, modified Mediterranean Diet Index; DS, Dietary Score; MAI, Mediterranean Adequacy Index; PREDIMED, PREvencion con DIeta MEDiterranea; NNS, Negev Nutrition Study; EPIC, European Prospective Investigation into Cancer and Nutrition; REGARDS, Reasons for Geographic and Racial Differences in Stroke; SUN, Seguimiento Universidad de Navarra; NOMAS, Northern Manhattan Study; HALE, Healthy Ageing: a Longitudinal study in Europe; NHS, Nurses Health Study; NIH-AARP Diet and Health Study, National Institutes of Health-AARP Diet and Health Study; MONICA, MONItoring trends and determinants of CArdiovascular disease. MI, myocardial infarction; CABG, coronary artery bypass grafting; PTCA, percutaneus transluminal coronary angioplasty; CVD, cardiovascular disease; CHD, coronary heart disease; ° In this study, the food group of cereals was not clearly defined (bread and potatoes); * With the exception of potatoes, the used dietary and t-MED scores had the same components in this study but were built differently.

**Table 2:** *A priori* dietary indexes and studies in which they have been evaluated or used to study the relationship between Mediterranean Diet and CVD, CHD, and cerebrovascular disease.

*A priori* Index	Authors (Year) (Reference)	Index Components	Score Range
MDS	Trichopoulou *et al.*, (1995) [4] Bilenko *et al.*, (2005) [10] ° Hoşcan *et al.*, (2015) [11] °	MDS	0–8
		8 components: M/S ratio; cereals (including bread and potatoes); vegetables; fruit; legumes; alcohol; meat and meat products; milk and dairy products	
Other Indexes adapted from the MDS: t-MED	Trichopoulou *et al.*, (2003) [12] Dilis *et al.*, (2012) [13] Misirli *et al.*, (2012) [14] Tsivgoulis *et al.*, (2015) [15] * Martínez-González (2011) [16] Gardener *et al.*, (2011) [17] * Agnoli *et al.*, (2011) [18] Turati *et al.*, (2015) [19]	t-MED	0–9
		9 components: M/S ratio; cereals (including bread and potatoes); vegetables; fruit and nuts; legumes; fish; alcohol; meat and meat products; milk and dairy products	
Other indexes adapted from the t-MED			
the score according to Knoops	Knoops *et al.*, (2004) [20]	the score according to Knoops	0–8
		8 components: M/S ratio; legumes, nuts and seeds; cereals; fruits; vegetables and potatoes; fish; meat and meat products; dairy products	
the Italian Mediterranean Index	Agnoli *et al.*, (2011) [18]	the Italian Mediterranean Index	0–11
		11 components: pasta; typical Mediterranean vegetables; fruit; legumes; olive oil; fish; soft drinks; butter; red meat; potatoes; alcohol	
a-MED	Fung *et al.*, (2009) [21] Mitrou *et al.*, (2007) [22]	a-MED	0–9
		9 components: M/S ratio; legumes; fruits; vegetables (excluding potatoes); nuts; whole grains; fish; red and processed meats; alcohol	
r-MED	Buckland *et al.*, (2009) [23]	r-MED	0–18
		9 components: Fruit, nuts, seeds (excluding fruit juices); vegetables (excluding potatoes); legumes; cereals; fish, sea foods; olive oil; meat and meat products, dairy products, alcohol	
m-MED	Trichopoulou *et al.*, (2005) [35] Hoevenaar-Blom *et al.*, (2012) [24]	m-MED	0–9
		9 components: M + P/S ratio; vegetables; legumes; fruit; cereals; fish; meat; dairy products; alcohol	
Other indexes adapted from the m-MED			
the score according to Sjögren	Sjögren *et al.*, (2010) [25]	the score according to Sjögren	0–8
		8 components: P/S ratio; vegetables and legumes, fruit; cereals and potatoes; fish; meat and meat products; milk and milk products; alcohol	
the scores according to Tognon	Tognon *et al.*, (2012) [26]	the score according to Tognon (2012)	0–8
		8 components: M + P/S ratio; vegetables and potatoes; fruit and juices; whole grain cereals; fish and fish products, meat and meat products; dairy products; alcohol	
	Tognon *et al.*, (2014) [27]	the score according to Tognon (2014)	0–8
		8 components: M + P/S ratio; vegetables; fruit; cereal grains; fish and fish products; meat, meat products and eggs; dairy products; alcohol	
DS	Pitsavos *et al.*, (2005) [5] Panagiotakos *et al.*, (2015) [28] Panagiotakos *et al.*, (2006) [29] Kastorini *et al.*, (2011) [30] Kastorini *et al.*, (2012) [31]	DS	0–55
		11 components: olive oil; whole grains; fruit; vegetables; potatoes; legumes; fish; meat and meat products; poultry; full fat dairy; alcohol	
MAI	Alberti-Fidanza *et al.*, (1999) [6] Fidanza *et al.*, (2004) [32] Menotti *et al.*, (2012) [33]	MAI	0.6–11.6 #
		18 components: bread; cereals; legumes; potatoes; vegetables; fresh fruit; nuts; fish; wine; vegetable oils; milk; cheese; meat; eggs; animal fat and margarines; sweet beverages; cakes, pies, cookies; sugar	
*a priori* Mediterranean Dietary Pattern	Martínez-González *et al.*, (2002) [7]	*a priori* Mediterranean Dietary Pattern	0–40
		8 components: olive oil; fiber; fruit; vegetables; fish; alcohol; meat and meat products; bread, pasta and rice	
PREDIMED score	Schröder *et al.*, (2011) [8] Estruch *et al.*, (2013) [34]	PREDIMED score	0–14
		14-componennts: olive oil; vegetables; fruit (including natural fruit juices); red meat and meat products; animal fats; sugar sweetened beverages; red wine; legumes; fish and shell fish; sweets and pastries; nuts; sofrito; white meat; olive oil as main culinary fat	

MDS, Mediterranean Diet Score; t-MED, Trichoupoulou Mediterranean Diet Index; a-MED, alternate Mediterranean Diet Index; r-MED, relative Mediterranean Diet Index; m-MED, modified Mediterranean Diet Index; DS, Dietary Score; MAI, Mediterranean Adequacy Index; PREDIMED, PREvencion con DIeta MEDiterranea; CVD, cardiovascular disease; CHD, coronary heart disease; M, monounsaturated fat; P, polyunsaturated fat; S, saturated fat; ° In this study, the food group of cereals was not clearly defined (bread and potatoes); * With the exception of potatoes, the used dietary and t-MED scores had the same components in this study but were built differently; # Score range among 16 cohorts of the Seven Countries Study (Fidanza *et al*.) [32].

## 4. Review

### 4.1. The Mediterranean Diet Score (MDS)

The MDS was the first index used to study adherence to the Mediterranean Diet in the population and it was developed by Trichopoulou *et al*. in 1995 [4]. It was based on eight main characteristics: monounsaturated fats (MUFAs) (from olive oil): saturated fats (SFAs) ratio; cereals (with inclusion of bread and potatoes); vegetables; fruit and nuts; legumes; alcohol; meat and meat products; and milk and dairy products. The score did not take into account sugars and syrups since, at that time, they had not been documented as dangerous for the health except for the total energy intake. The gender-specific median value was used as a cut-off point for each component. The hypothesis to be verified was that a diet with a high intake of cereals, vegetables, fruit, olive oil and legumes, a low intake of meat and dairy and a moderate intake of alcohol was healthy. The index was applied to elderly people, inhabitants of three rural Greek villages, who still followed the traditional Mediterranean Diet, with the aim of evaluating the relationship between adherence to diet and overall mortality [4].

The MDS was then used to examine the adherence to Mediterranean Diet and its relationship with coronary heart disease (CHD) in a random sample of 1159 Jewish people in Israel. In men, the odds ratio (ORs) for myocardial infarction, coronary bypass, angioplasty and any other cardiovascular disease (CVD) were 1.23 (*p* = 0.04), 1.56 (*p* = 0.01), 1.42 (*p* = 0.003), and 1.28 (*p* < 0.01), respectively, for each decrease in the MDS (score 0–8) in the logistic regression models adjusted for hypertension, hypercholesterolemia, diabetes mellitus, age, education, body mass index, and place of birth. A similar trend was observed in women, but no statistically significance was evident. In this study the mean consumption of olive oil was 0.82 g per day per person and the major contributors to MUFAs were canola oil, sunflower oil and dairy [10].

Very recently, the MDS was applied to a population study of 900 Turkish people in Alanya. After 5.1 years of follow-up, men with lower adherence to the Mediterranean Diet (score 0–4) had a higher risk of CHD morbidity (multivariate adjusted OR: 2.2; 95% CI (confidence interval): 1.03, 3.9; *p* = 0.03) compared to men with higher adherence (score 5–8). No association was found in women. In this study CHD was defined as myocardial infarction or coronary bypass or coronary angioplasty. Note that fish consumption was very low (2%) in this cohort and the consumption of olive oil was 0.9 g per day per person; the majors contributors to MUFAs were corn and sunflower oils [11].

In 2003 the MDS was updated by Trichopoulou *et al*. with the inclusion of another component, a moderate fish consumption and, just like in the previous study [4], each component received a value of 0 or 1 using the cut-off value of the gender-specific median. Subjects were assigned a value of 0 if the consumption of components considered beneficial (vegetables, legumes, fruit and nuts, cereals, fish, MUFAs:SFAs ratio) was below the median, whereas individuals were assigned a value of 1 if they had a consumption of beneficial food at or above the median. Otherwise, people with consumption of components considered harmful (meat, poultry, and dairy products) below the median were assigned a value of 1, whereas people with consumption at or above the median were assigned a value of 0. With regards to alcohol, a value of 1 was assigned to subjects consuming a moderate amount (*i.e*., between 10 and 50 g per day for men and between 5 and 25 g per day for women) and a value of 0 otherwise. Therefore, this MDS with fish (t-MED) ranged from 0 (minimal adherence to Mediterranean Diet) to 9 (maximal adherence to Mediterranean Diet) [12]. In the Greek cohort of the *European Prospective Investigation into Cancer and Nutrition* (EPIC) study, 22,043 adults were followed for a median of 44 months, examining the relationship among diet and total mortality, CHD mortality and cancer mortality. A two-point increase in the t-MED was associated with a reduction in CHD mortality by 33% (multivariate adjusted hazard ratio (HR): 0.67; 95% CI: 0.47, 0.94) [12]. A contemporary study showed that dietary habits resembling the traditional Mediterranean Diet were widespread in Greece [36].

The Greek EPIC cohort has been followed in relation to CHD [13] and cerebrovascular disease [14]. During a median period of 10 years, a two-point increase in the t-MED was associated with a decrease in CHD mortality by 22% (multivariate adjusted HR: 0.78; 95% CI: 0.66, 0.92; *p* for trend = 0.003) and a non-significant reduction in CHD incidence (multivariate adjusted HR: 0.92; 95% CI: 0.84, 1.02; *p* for trend = 0.115) [13]. A two-point increase in t-MED was inversely associated with cerebrovascular disease incidence (multivariate adjusted HR: 0.85; 95% CI: 0.74, 0.96) during a median period of 10.6 years. In the subgroups analyzed by gender, the association was significant in women (multivariate adjusted HR: 0.81; 95% CI: 0.67, 0.98) but not in men. Overall, the protective effect of adherence to the Mediterranean Diet, evaluated by the t-MED, was evident in the incidence of ischemic (multivariate adjusted HR: 0.54; 95% CI: 0.29, 1.01; *p* for trend = 0.048) but not on the incidence of hemorrhagic cerebrovascular disease. The association between t-MED and cerebrovascular disease mortality was not significant [14].

Very recently, the t-MED was evaluated in 20,197 subjects enrolled in the *Reasons for Geographic and Racial Differences in Stroke* (REGARDS) study. In this population-based sample of US black and white adults, during a mean follow-up period of 6.5 years, a higher adherence to the Mediterranean Diet (score 5–9) was associated with a lower risk of incident ischemic stroke in comparison with a lower adherence (score 0–4) (multivariate adjusted HR: 0.79; 95% CI: 0.65, 0.96; *p* = 0.016). A one-point increase in t-MED was independently associated with a 5% reduction in the risk of incident ischemic stroke (95% CI: 0%, 11%). No association was found between incident hemorrhagic stroke and t-MED [15]. With the exception of potatoes, the used dietary and the t-MED scores had the same components in this study but they were built differently.

The t-MED, applied to the *Seguimiento Universidad de Navarra* (SUN) study, a Spanish cohort of 13,609 young graduates, followed for a median of 4.9 years, was inversely associated with CVD and CHD. CVD was a composite outcome of myocardial infarction mortality, stroke mortality, acute coronary syndromes, revascularization procedures, and stroke. A higher adherence to the Mediterranean Diet (score 7–9) was associated with a lower risk of developing CVD (multivariate adjusted HR: 0.41; 95% CI: 0.18, 0.95; *p* for trend = 0.07) and CHD (multivariate adjusted HR: 0.42; 95% CI: 0.16, 1.11; *p* for trend = 0.04). A two-point increase in the t-MED was associated with a 20% decrease in CVD risk (multivariate adjusted HR: 0.80; 95% CI: 0.62, 1.02) and a 26% reduction in CHD risk (multivariate adjusted HR: 0.74; 95% CI: 0.55, 0.99). It is noteworthy that using a t-MED built without cereals, namely a score with eight components, the inverse association between adherence to the Mediterranean Diet and CVD became significant (*p* for trend = 0.03) and the negative relationship between adherence to the Mediterranean Diet and CHD became more evident (*p* for trend = 0.01). Since both refined cereals and whole grains are considered a unique category in the t-MED, the authors hypothesized a negative impact from refined cereals on CVD risk [16]. Refined cereals have a negative impact on CVD risk because they raise the diet glycemic load, which has been associated with a decrease in HDL cholesterol, increase in fasting plasma triglycerides and fasting insulin, promotion of oxidative stress, low-grade systemic inflammation, and procoagulant activity [37].

In the *Northern Manhattan Study* (NOMAS), a multiethnic cohort of 2568 subjects followed for a mean period of 9 years, the association between adherence to Mediterranean Diet (estimated by the t-MED) and CVD events was not statistically significant for ischemic stroke and myocardial infarction but it was for vascular death (multivariate adjusted HR: 0.71; 95% CI: 0.49, 1.04; *p* for trend = 0.04 between the highest and the lowest quintile of the t-MED). A one-point increase in the t-MED was associated with a 9% decrease of the risk of vascular death (multivariate adjusted HR: 0.91; 95% CI: 0.85, 0.98; *p* for trend < 0.05). Consumption of alcohol, fish and legumes were the components driving the inverse association of t-MED with vascular death [17]. Also in this study, with the exception of potatoes, the used dietary and the t-MED scores had the same components but were built differently.

The t-MED applied to the Italian cohort of the *long-tErm follow-up of antithrombotic management Patterns In acute CORonary syndrome patients* (EPICOR) study involving 40,681 volunteers recruited from five cities uniformly distributed in the Italian territory and followed for a mean of 7.9 years was inversely associated with the risk of ischemic stroke, but the trend among the tertiles of intake was not significant. The association with the risk of hemorrhagic stroke was positive without a statistic significance [18].

In a hospital-based case-control study performed in a northern Italian City, 760 patients with a first episode of non-fatal myocardial infarction were matched with 682 controls by gender and age, admitted to the hospital for non-neoplastic conditions, unrelated to known risks for myocardial infarction or dietary modification. Adherence to the Mediterranean Diet was assessed by the t-MED. The risk of a first episode of myocardial infarction was reduced by 45% (multivariate adjusted OR: 0.55; 95% CI: 0.40, 0.75; *p* for trend < 0.01) in subjects with higher adherence to the Mediterranean Diet (score ≥ 6) as compared to subjects with lower adherence (score < 4). A one-point increase in the t-MED was associated with a reduced risk of a first episode of myocardial infarction by 9% (multivariate adjusted OR: 0.91; 95% CI: 0.85, 0.98). High consumption of vegetables and legumes were inversely associated with non-fatal myocardial infarction risk [19].

A modification of the t-MED was proposed by Trichopoulou *et al*. in 2005, with the aim of making the application of t-MED to non-Mediterranean populations, in which the intake of MUFAs from olive oil was minimal, possible. The modified t-MED (m-MED) was achieved by replacing the MUFAs:SFAs ratio with the MUFAs + Polyunsaturated fats (PUFAs): SFAs ratio [35].

The m-MED was applied to the Dutch cohort of the EPIC study, EPIC-NL, that included 34,708 participants followed for a mean of 11.8 years. A two-point increase in the m-MED was inversely and significantly associated with fatal CVD (multivariate adjusted HR: 0.78; 95% CI: 0.69, 0.88), composite CVD (fatal CVD, non-fatal myocardial infarction, non-fatal stroke) (multivariate adjusted HR: 0.85; CI 95%: 0.80, 0.91), incident myocardial infarction (multivariate adjusted HR: 0.86; 95% CI: 0.79, 0.93), incident stroke (multivariate adjusted HR: 0.88; 95% CI: 0.78, 1.00) and pulmonary embolism (multivariate adjusted HR: 0.74; 95% CI: 0.59, 0.92). No association was found between m-MED and incident angina pectoris, transient ischemic attack and peripheral arterial disease. Interestingly, the association of m-MED to fatal CVD, CVD incidence, composite CVD and myocardial infarction was mostly mitigated when alcohol was excluded from m-MED [24].

In the literature, we identified four variants of t-MED—the score according to Knoops *et al*. [20], the alternate Mediterranean Diet Index (a-MED) [38], the relative Mediterranean Diet Index (r-MED) [23], and the Italian Mediterranean Index [18]—and three variants of m-MED: the score according to Sjögren *et al.* [25] and the scores according to Tognon *et al.* [26,27].

In the *Healthy Ageing: a Longitudinal study in Europe* (HALE) study, performed in 2339 elderly people of 11 European countries, Knoops *et al*. used a modified t-MED based on eight components: MUFAs:SFAs ratio; legumes, nuts and seeds; cereals; fruit; vegetables and potatoes; fish; meat and meat products; and dairy products. The intake of each component was adjusted to daily intakes of 2500 kcal for men and 2000 kcal for women. For the first six components considered to be healthy, a value of 1 was assigned to people whose consumption was at least as high as the gender-specific median value, and a value of 0 to the others. Reverse values were assigned to the last two components considered unhealthy. Alcohol was evaluated as a separate lifestyle factor since many studies observed an independent effect of alcohol on survival. The score ranged from 0 (minimal adherence to the Mediterranean Diet) to 9 (maximal adherence to the Mediterranean Diet). A score of at least four points was associated with lower CHD mortality (multivariate adjusted HR: 0.61; 95% CI: 0.43, 0.88) and CVD mortality (multivariate adjusted HR: 0.71; 95% CI: 0.58, 0.88). The combination of four healthy lifestyles (high adherence to the Mediterranean Diet, moderate alcohol intake, moderate-high physical activity levels, nonsmoking) reduced the CHD mortality and the CVD mortality more than 50% in comparison with none or one healthy lifestyle [20].

The a-MED is a t-MED modified by excluding potatoes from the vegetable group, separating fruit and nuts into two groups, eliminating the dairy group, including whole-grain products only, including only red and processed meats for the meat group, and assigning alcohol intake between 5 and 15 g per day for 1 point. These changes were based on dietary patterns that were consistently associated with lower risk of chronic disease in clinical and epidemiological studies. The a-MED ranged from 0 to 9 [38]. In the *Nurses Health Study* (NHS) a cohort of 74,886 female nurses followed for 20 years, the risk of total (non-fatal and fatal cases) CHD was lower in the highest quintile of the a-MED compared with the lowest quintile (multivariate adjusted relative risk (RR): 0.71; 95% CI: 0.62, 0.82; *p* for trend < 0.0001). Total stroke (non-fatal and fatal cases) was lower in the highest quintile compared with the lowest quintile of a-MED (multivariate adjusted RR: 0.87; 95% CI: 0.73, 1.02; *p* for trend = 0.03). In the a-MED, alcohol intake could result from regular consumption of beer, spirits or wine while MUFAs resulted mainly from meat and only minimally from olive oil [21].

In the *National Institutes of Health-AARP Diet and Health Study cohort*, consisting of 380,296 people followed for 5 years, the risk of mortality for CVD was significantly lower in men and women with a higher adherence to the Mediterranean Diet (score 6–9) compared to those with a lower adherence (score 0–3), evaluated by the a-MED (men: multivariate adjusted HR: 0.78; 95% CI 0.69, 0.87; *p* for trend < 0.001; women: multivariate adjusted HR: 0.71; 95% CI: 0.68, 0.97; *p* for trend = 0.01) [22].

The r-MED was used to evaluate the exposure to the Mediterranean Diet in the Spanish cohort of EPIC. It derived from t-MED and consisted of nine components. Among these, six components were considered typical of Mediterranean diet: fruit (including nuts and seeds but excluding fruit juices); vegetables (excluding potatoes); legumes; cereals (whole and refined grains); fresh fish and sea foods; and olive oil. Two components were considered not typical of the Mediterranean Diet: meat and meat products; and dairy products (including low-fat and high-fat milk, yogurt, cheese, cream desserts, and dairy and nondairy creams). Each component, except alcohol, was measured as grams per 1000 kcal/day and was divided into tertiles of dietary intakes. A value of 0, 1 and 2 to the first, second and third tertiles of intake, respectively was assigned to typical components. Non-typical components were assigned with an inverted score. Alcohol was scored as a dichotomous variable and 2 points was assigned for an intake from 5 to 25 g per day for women and from 10 to 50 g per day for men. Intakes above or below these ranges were scored 0 points. Then, the theoretical r-MED ranged between 0 (no adherence) to 18 (maximal adherence). In the multivariate analysis performed on data for 40,757 subjects of the cohort, followed for a mean of 10.4 years, the risk of fatal and non-fatal (myocardial infarction or unstable angina requiring revascularization) incident CHD was lower in subjects with higher adherence to the Mediterranean Diet (score 11–18) compared to subjects with lower adherence (score 0–6) (multivariate adjusted HR: 0.60; 95% CI: 0.47, 0.77; *p* for trend < 0.001). A one-point increase in the r-MED was associated with a 6% lower risk of CHD (multivariate adjusted HR: 0.94; 95% CI: 0.91, 0.97; *p* for trend < 0.001). The use of t-MED instead of r-MED was associated with an almost identical decrease in CHD risk for a two-point increase in both scores [23].

The *Italian Mediterranean Index* was developed to adapt the t-MED to Italian eating behavior [18]. It consisted of 11 components: six typical Mediterranean food (pasta; typical Mediterranean vegetables; fruit; legumes; olive oil; and fish), four non-Mediterranean foods (soft drinks; butter; red meat; and potatoes), and alcohol. People whose consumption of typical Mediterranean foods was in the 3rd tertile of distribution received 1 point whereas all others received 0 points. People whose consumption of non-Mediterranean foods was in the first tertile of the distribution, received 1 point, and all others received 0 points. For alcohol, people whose consumption was up to 12 g per day received 1 point, whereas abstainers and people whose consumption was >12 g per day received 0 points. The theoretical score ranged from 0 to 11. This score applied to 40,681 subjects of EPICOR study, followed for a mean of 7.9 years, was inversely associated with ischemic stroke (multivariate adjusted HR: 0.37; 95% CI: 0.19–0.70; *p* for trend = 0.001 ) and hemorrhagic stroke (multivariate adjusted HR: 0.51; 95% CI: 0.22–1.20; *p* for trend = 0.07 ) [18].

The score according to Sjögren *et al*. was a variant of the m-MED and was applied to a population-based longitudinal study of 924 Swedish men. Due to a very low intake, nuts and seeds were excluded by the score and leguminous plants were pooled with vegetables. PUFAs replaced MUFAs because the consumption of the olive oil in this population was very low and SFAs and MUFAs had similar food origins and therefore strongly correlated. During a mean follow-up of 10 years, a higher adherence to the Mediterranean Diet (score 6–8) was associated with a lower risk of CVD mortality by 81% (multivariate adjusted HR: 0.19; 95% CI: 0.04, 0.86; *p* for trend = 0.009) as compared to lower adherence (score 0–2) [25].

The score according to Tognon *et al*. was based on eight components: vegetables and potatoes; fruit and juices; whole grain cereals; fish and fish products; MUFAs + PUFAs:SFAs ratio; alcohol intake; meat and meat products; and dairy products. The cut-off points were the gender-specific medians and the value of 0 was assigned to people whose consumption was under the gender-specific median for the first six components and above for the last two components. The value of 1 was assigned to people whose consumption was above the gender-specific median for the first six components and under the gender-specific median for the last two components. The final score varied from 0 (low adherence) to 8 (high adherence) [26]. In a population based study performed in Västerbotten, a North Sweden County with 77,151 subjects followed for 17 years, the score was significantly associated, only in women and not in men, with CVD mortality (multivariate adjusted HR: 0.90; 95% CI: 0.82, 0.99; *p* for trend < 0.05), and with mortality for myocardial infarction (multivariate adjusted HR: 0.84; 95% CI: 0.71, 0.99; *p* for trend < 0.05). The mortality for stroke was not associated with the Mediterranean Diet in men and women. Only alcohol intake was independently and inversely associated with CVD mortality among the eight components of the score [26].

A score that was very similar to the previous one [26] was used to evaluate the relationship between Mediterranean Diet and CVD in a Danish cohort of the *MONItoring trends and determinants of Cardiovascular disease* (MONICA) project [27]. It was based on eight components: MUFAs + PUFAs: SFAs ratio; alcohol intake; vegetables; fruit; cereal grains; fish and fish products; meat, meat products and eggs; and dairy products. The cut-off points were the gender-specific medians and the value of 0 was assigned to people whose consumption was under the median for the first six components and above for the last two components. The value of 1 was assigned to people whose consumption was above the median for the first six components and under the median for the last two components. Two different procedures were used to produce two different scores. The first procedure excluded mixed dishes (score 1), and the second included ingredients extrapolated from mixed dishes or recipes (score 2). A third score was created in the same way as score 2 except considering wine instead of total alcohol intake (score 3). None of the scores were associated with stroke mortality and incidence (fatal and non-fatal cases) in the multivariate analysis. The score 1 was inversely associated with CVD mortality and incidence (fatal and non-fatal cases) and with myocardial infarction but without statistical significance. The score 2 was associated with CVD incidence (fatal and non-fatal cases) (multivariate adjusted HR: 0.94; 95% CI:0.89, 0.99; *p* for trend < 0.05), CVD mortality (multivariate adjusted HR: 0.90; 95% CI: 0.82, 0.99; *p* for trend < 0.05), myocardial infarction incidence (fatal and non-fatal cases) (multivariate adjusted HR: 0.89; 95% CI: 0.80, 1.00; *p* for trend < 0.05), and myocardial infarction mortality (multivariate adjusted HR: 0.80; 95% CI 0.67, 0.96; *p* for trend < 0.05). Score 3 was associated with the same outcomes slightly more than score 2 [27].

### 4.2. The Dietary Score (DS)

The DS, proposed in 2005 and inspired by the dietary guidelines of the Greek Mediterranean Diet pyramid for adults [39], was directly associated with antioxidant capacity and inversely correlated with oxidized LDL-cholesterol serum concentrations, in a random sample of healthy adults in the *ATTICA* study [5]. It consisted of 11 components: whole grains; fruit; vegetables; potatoes; legumes; olive oil; fish; meat and meat products; poultry; full fat dairy; and alcohol. With the exclusion of alcohol, the frequency of intakes was categorized as never, rare (1–4 servings per month), frequent (5–8 servings per month), very frequent (9–12 servings per month), weekly (13–18 servings per month) and daily (>18 servings per month). For these intake frequencies a value of 0, 1, 2, 3, 4, and 5 respectively was assigned for the first seven components. The values were reversed for frequency of intakes of red meat, poultry, and full fat dairy foods. The alcohol intake was scored 5 if <300 mL per day of wine, 0 for consumption >700 mL per day or none, and scored 4, 3, 2, 1, for intakes of 300–400, 400–500, 500–600, and 600–700 mL per day, respectively. It was stated that 100 mL of wine contained 12 g of alcohol. The theoretical range of the score varied from 0 to 55. The adherence to the Mediterranean Diet decreased the CVD risk in the *ATTICA* study that involved 2583 participants: during 10 years of follow-up, a one-point increase in the score decreased the risk by 4% (multivariate adjusted RR: 0.96; 95% CI: 0.93, 1.00) independently of socio-demographic variables, lifestyle, and clinical factors. The protective effect of Mediterranean Diet was also evident in participants at risk such as smokers, sedentary individuals, and obese people. No component of the dietary score was significantly associated with CVD risk, in line with the hypothesis that the Mediterranean pattern acts as a whole [28].

This score was also applied in some case-control studies.

In the *CARDIO2000*, a case-control study, 848 patients who had been hospitalized for a first symptom of coronary heart disease were matched with 1078 controls by age, gender and the region they came from. The subjects in the highest tertile of the score had a reduced odds of having acute coronary syndromes by 46% (multivariate adjusted OR: 0.54; 95% CI: 0.44, 0.66) compared with subjects in the lowest tertile. An 11/55-unit increase in DS was associated with a reduced odds of having acute coronary syndromes by 27% (multivariate adjusted OR: 0.73; 95% CI: 0.66–0.89) [29].

In a case-control study of 250 consecutive patients with a first episode of acute coronary syndrome and 250 consecutive patients with a first ischemic stroke, matched with 500 healthy subjects by gender and age, a one-point increase in the score reduced the odds of having acute coronary syndrome (multivariate adjusted OR: 0.91; 95% CI: 0.87, 0.96) and ischemic stroke (multivariate adjusted OR: 0.88; 95% CI: 0.82, 0.94) [30].

In a case-control study of 250 consecutive patients with a first ischemic stroke matched with 250 controls by gender, age and region, a one-point increase in DS reduced the odds of having a first ischemic stroke by 17% (multivariate adjusted OR: 0.83; 95% CI: 0.72, 0.96) in non-hypercolesterolemic participants and by 10% (multivariate adjusted OR: 0.90; 95% CI: 0.81, 0.99) in hypercolesterolemic participants [31].

### 4.3. The Mediterranean Adequacy Index (MAI)

The MAI is a dietary score built taking into account the Mediterranean Diet of Nicotera as a reference. In the early 1960s this diet was very similar to the Corfu and Crete diets [6], with low CHD mortality rate at 25-year of follow-up of the *Seven Countries Study* [40]. This score, consisting of 18 food or food groups, is computed by dividing the sum of the total energy percentages of the food groups typical of the reference Mediterranean Diet (bread; cereals; legumes; potatoes; vegetables; fresh fruit; nuts; fish; wine; and vegetable oil) by the sum of the total energy percentages of the food groups less typical of the reference Mediterranean Diet (milk; cheese; meat; eggs; animal fats and margarines; sweet beverages; cakes, pies, cookies; and sugar). The higher the MAI, the greater the amount of energy derived from typical Mediterranean foods [6]. The MAI could be expressed as g per day, or g per 1000 kcal, or g per 4.2 MJ [32].

The MAI, computed in random samples of men surveyed for their eating habits and belonging to 16 cohorts of the *Seven Countries Study*, was inversely associated with the 25-year death rates from CHD (*r* = −0.72; *p* = 0.001) [32]. The HR for 1 unit of natural log of MAI (approximately corresponding to 2.7 units of MAI) was associated with a CHD mortality decrease of 26% (multivariate adjusted RR: 0.74; 95% CI: 0.55, 0.99) in 20 years of follow-ups and of 21% (multivariate adjusted RR: 0.79; 95% CI: 0.64, 0.97) in 40 years of follow-ups in two Italian rural cohorts of the *Seven Countries Study,* Crevalcore and Montegiorgio. The statistical analysis was multivariate adjusted for the covariates [33].

### 4.4. A Priori Mediterranean Dietary Pattern

This score was applied to a case-control study of 171 patients with a first myocardial infarction matched with 171 controls by gender and age, hospitalized for diseases considered not to be related to the diet. It is based on six food groups or nutrients considered as protective, and two groups of foods of the Mediterranean Diet considered harmful. The first consisted of: olive oil; fibers; fruit; vegetables; fish; and alcohol. For each of these items the distribution according to quintiles within the study was calculated and each subject received points from 1 to 5 corresponding to the quintile of intake from the lowest to the highest quintile. The latter consisted of: meat and meat products; foods with high glycemic load as white bread, pasta and rice. For these components each subject received 1 for the highest quintile and 5 for the lowest quintile. For each subject the score was obtained summing the eight quintiles values. The theoretical score ranged between 0 and 40. This score was applied to 342 subjects of the study. The subjects with score ≥30 had a risk of a first myocardial infarction lower of 79% (multivariate adjusted OR: 0.21; 95% CI: 0.06, 0.73; *p* for trend = 0.01) compared with subjects scored <20. The risk was reduced by 8% for a one-point increase in the score (multivariate adjusted OR: 0.92; 95% CI: 0.86, 0.98; *p* for trend = 0.01) [7].

### 4.5. The (PREDIMED) Score

The *PREDIMED* is a Spanish multi-center trial of 7447 people at high risk for CVD, randomly assigned to one of three groups: participants that received advise to reduce dietary fat (control diet); participants that received advice on Mediterranean Diet and provision of extra-virgin olive oil (approximately 1 liter per week); participants that received advice on Mediterranean Diet and provision of mixed nuts (30 g per day: 15 g of walnuts, 7.5 g of hazelnuts, and 7.5 g of almonds) [34]. The adherence to the Mediterranean Diet was evaluated at baseline and during the study by a 14-point Mediterranean Diet Adherence Screener (MEDAS). It consists of 12 questions on food intake frequency and two questions on dietary habits characteristic of the Spanish Mediterranean Diet [8]. A value of 1 point was assigned if these criteria were met:

- ≥4 tablespoons of olive oil per day (including that used in frying, salads *etc*.) (1 tablespoon = 13.5 g
- ≥2 servings of vegetables per day (at least 1 portion raw or as salad) (1 serving = 200 g)
- ≥3 fruit units (including natural fruit juices) per day
- <1 serving of red meat or meat products (1 serving =100–150 g) per day
- <1 serving of animal fat per day (1 serving = 12 g)
- <1 cup of sugar-sweetened beverage per day (1 cup = 100 mL)
- ≥7 glasses of red wine per week
- ≥3 servings of legumes per week (1 serving = 150 g)
- ≥3 servings of fish or shellfish per week (1 serving: 100–150 g fish, or 4–5 units, 200 g shellfish)
- <3 commercial sweets or pastries per week (not homemade)
- ≥3 servings of nuts (including peanuts) per week
- ≥2 times per week of a dish with a traditional sauce of tomatoes, garlic, onion, or leeks sautéed in olive oil
- olive oil as main culinary fat
- preferential consumption of chicken, turkey, rabbit meat instead of veal, pork, hamburger or sausage

A value of 0 point was assigned if these criteria were not met. The final PREDIMED score ranged from 0 to 14.

The participants in the three groups at the beginning of the study had a similar score. During the study, the score of participants in the Mediterranean Diet group increased in comparison with the control group, and after three years the differences were significant for 12 out of 14 components of the score. An evaluation of biomarkers (urinary hydroxytyrosol in the group receiving extra-virgin oil, plasma alpha-linolenic acid levels in the group receiving nuts) confirmed the compliance to dietary advice. After a median follow-up of 4.8 years, the rate of major CVD events (myocardial infarction, stroke, death for CVD) was reduced by 30% (multivariate adjusted OR: 0.70; 95% CI: 0.54, 0.92; *p* = 0.01) in the group assigned to the Mediterranean Diet with extra-virgin olive oil and by 28% (multivariate adjusted OR: 0.72; 95% CI: 0.54, 0.96; *p* = 0.03) in the group assigned to the Mediterranean Diet with nuts, compared with the control group. In the subgroup analysis, the supplemented Mediterranean Diet had a clear protective effect on stroke (multivariate adjusted OR: 0.61; 95% CI: 0.44, 0.86; *p* = 0.005) but the protective effect on myocardial infarction and CVD death in comparison with the control diet did not reach statistical significance [34].

### 4.6. The Mediterranean-Style Dietary Pattern Score (MSDPS)

The MSDPS was elaborated by Rumawas *et al.* [9] using the Greek Mediterranean Diet pyramid for adults as a reference [39] and was based on 13 components corresponding to 13 food groups of the pyramid: whole grains; fruit; vegetables; milk and dairy products; wine; fish; poultry; olives, legumes, nuts; potatoes; eggs; sweets; meat; and olive oil. With the exception of olive oil, each group was scored from 0 to 10 depending on the compliance to the numbers of servings suggested in the pyramid. A penalty was assigned for a possible overconsumption by subtracting a point proportionally to the number of servings consumed that exceeded the recommended intake for the considered food group. If the score became negative it was defaulted to zero. Olive oil was scored 10 if its use was exclusive, 5 if its use was along with other vegetable oils, 0 for no use. For each subject the MSDPS was calculated by summing the values of 13 components, and dividing this sum by the theoretical maximum sum of 130 and multiplying by 100 by the aim to obtain a scale of standardized values ranging from 0 to 100. In view of the mixture of Mediterranean and non-Mediterranean foods that real patterns have, the previous score was corrected by a continuous factor ranging from 0 to 1 depending on the proportion of energy intake derived by foods not included in the pyramid. The MSDPS was applied to dietary data collected during the 7th examination of the Framingham Offspring Cohort. The quintiles of MSDPS were significantly and positively associated with the dietary intakes of fiber, *n*-3 PUFAs (linolenic acid, eicosapentaenoic acid, docosahexaenoic acid), antioxidant vitamins (β-carotene, folate, vitamin C, vitamin E, lycopene), calcium, magnesium, potassium, and inversely and significantly with added sugars, glycemic index, SFAs, trans-fat acids, *n*-6 PUFAs (linoleic acid and arachidonic acid): *n*-3 PUFAs ratio, MUFAs, and oleic acid. The inverse association of MSDPS with MUFAs and oleic acid depended on a large intake of meat (including poultry). The authors concluded that the MSDPS was a useful tool to evaluate the adherence to traditional Mediterranean Diet in a non-Mediterranean population [9].

We could not find any study investigating the relationship between Mediterranean Diet and CVD risk using this score.

## 5. Critical Appraisal

From the analysis of the above data it is evident that all the studies show a protective effect of Mediterranean Diet on CVD. Nevertheless the protective effect of the Mediterranean Diet against CHD and stroke is very different across the studies. Are there different types of Mediterranean Diets with different protective effects on these outcomes?

Two questions should be taken into consideration: Which Mediterranean Diet is the object of evaluation? How was exposure to the diet measured?

In most of the studies mentioned above, the score of dietary exposure was not established using traditional Mediterranean Diet of the early 1960s as a reference, and there are major deviations from the early dietary pattern that provides the best scientific evidence of a protective role against CVD [40,41]. The divergences observed are both qualitative and quantitative.

### 5.1. Qualitative Score Divergences from Traditional Mediterranean Diet

Fidanza *et al*. described the diet of Nicotera, a rural small town in the Calabria Region in Southern Italy. It was a cohort of the *Seven Countries Study,* but because of both a shortage of funds and similarity with the two cohorts of Greece, Creta and Corfu, it was not followed longitudinally [42]. The various components of the Nicotera diet, evaluated using the weighed method for seven days in three different seasons of 1960 and expressed as percentages of caloric intake provided, were the following: cereals (50%–59%), extra virgin olive oil (13%–17%), vegetables (2.2%–3.6%), potatoes (2.3%–3.6%), legumes (3%–6%), fruit (2.6%–3.6%, including nuts representing about 3% of the weight of all fruit), fish (1.6%–2.0%), and red wine (1%–6%). Meat (2.6%–5.0%), dairy products (2%–4%), eggs and animal fats were rarely eaten [31]. The bread was homemade from stone ground wheat [42] and was sourdough leavened [43].

Another source of dietary epidemiological data is that provided by the *Etude des consommations alimentaires des populations de onze regions de la communauté europeenne en vue de la determination des niveaux de contamination radioactive* (EURATOM), a study carried out in the early 1960s in 11 areas of seven European countries. Two areas were in Southern Italy, in Campania and Basilicata. The study was very accurate, using the weighed inventory method over seven consecutive days [44]. Here, the intake of pasta and bread was about 490 g per day providing 60% of total energy intake, the intake of fruit and vegetables was 426 g per day providing 9% of total energy intake, the intake of meat and fish was 62 g per day providing 5% of total energy intake, the intake of fats and oils was 51 g per day providing 18% of total energy intake, the intake of milk and dairy products was 87 g per day providing 4% of total energy intake, the intake of sugar was 15 g per day providing 2% of total energy intake, the intake of sweets was 1 g per day. The fiber intake was 24.4 g per day and 60% was derived from wheat while the remaining part came from tomatoes, onions, artichokes, pulses, eggplants and fruit. Added oils were almost exclusively vegetable oils, namely olive oil, whereas lard and butter were hardly ever used. Margarine was not used at all. The total of dietary lipids was 73 g per day, *i.e.*, 28% of total energy intake. Only the olive oil was used as vegetable oil and in quantity of 49 g per day. If it was considered only added lipids, the MUFAs:SFAs ratio was 3.9 while the PUFAs/SFAs ratio was 0.53. If it was considered total lipids the MUFAs:SFAs ratio was 2.29 while the PUFAs:SFAs ratio was 0.41 [45].

To sum up, the traditional Mediterranean Diet is well described by the Greek Mediterranean Diet pyramid for adults. It recommends a daily intake of 8 servings of whole grains, 3 servings of fruit, 6 servings of vegetables, 2 servings of dairy products, 3 glasses of wine for men and 1.5 glasses of wine for women; weekly intakes of 6 servings of fish, 4 servings of poultry, 4 servings of olives, pulses and nuts, 3 servings of potatoes, 3 serving of eggs, 3 servings of sweets; a monthly intake of 4 servings of red meat; and olive oil as the main added lipid [39].

Whole grains are, together with extra virgin olive oil and red wine, a peculiar characteristic of the Mediterranean Diet. The inclusion of whole grains into the score of dietary exposure to the Mediterranean Diet is consistent with the characteristics of traditional Mediterranean Diet but also with the scientific evidence of their protective role against CVD. In a pioneering study by Morris *et al*., a high intake of fiber from cereals was a protective factor for CHD incidence during 20 years of follow-up in 337 healthy English men. It was not evident that CHD was associated with refined cereals intakes [46]. In a recent exhaustive review of pooled/meta-analyses and systematic reviews, the protective effect of whole grains against CVD was higher than that of fruit and vegetables with a risk reduction by a maximum of 29%, 23% and 23% respectively, for the highest levels of consumption [47].

Whole grains have a recognized protective effect against CVD risk factors, such as type 2 diabetes mellitus [48,49,50,51,52,53], total and LDL cholesterol [54,55], hypertension [56,57,58], and low grade systemic inflammation [59,60]. Besides, a higher intake of whole grains has been associated with a better body weight [61,62,63,64]. Cereal fiber partly accounts for the protective effects against CVD mortality [65]. If whole grains cereals seem to be protective, refined cereals have either a neutral or harmful effect on CVD and other diet-related chronic diseases [47]. However a review of 135 relevant articles evaluating the relationship between consumption of refined cereal grains and health outcomes, shows that a consumption of up to 50% of all grain foods as refined grain foods (without high levels of added fat, sugar, or sodium) is not associated with increased CVD and other diet-related chronic diseases risk [66]. According to some authors whole grains products should be distinguished from refined food in the score of dietary exposure [67].

Seemingly, the substitution of MUFAs for olive oil in the indexes of Mediterranean Diet creates some concern since many healthy effects depend on minor components [68,69,70] and because of doubts raised by some authors on the cardio-protective effect of MUFAs [71]. Indeed, dietary MUFAs can come from other vegetable oils (high-oleic safflower and sunflower oils, canola oil), mostly nuts, peanuts, avocados, and from animal products (meat, eggs, lard) [72]. In a systematic review and meta-analysis of cohort studies, the comparison of the top *versus* bottom third of the distribution of a combination of MUFAs (of both plant and animal derived from), olive oil, oleic acid, and MUFAs:SFAs ratio resulted in a significant risk reduction for CVD mortality, CVD events, stroke and all-cause mortality. However, in subgroup analyses, only higher intakes of olive oil were associated with a significant decrease of risk for CVD events, stroke and all-cause mortality. By contrast, MUFAs of mixed animal and vegetable sources *per se* were not associated with these outcomes [73].

In the same way, the substitution of alcohol for wine in Mediterranean Diet indexes deserves to be evaluated as there is some evidence that wine offers greater CVD protection in comparison to other alcoholic beverages, possibly because of phenolic compounds [74,75,76]. The score proposed by Tognon *et al*. including wine instead of total alcohol intake was better associated with CVD outcomes [27].

The *a priori* indexes used to evaluate the adherence to the Mediterranean Diet should consider whole grains and refined grains, olive oil and MUFAs, and wine and alcohol in a different way. Whole cereal grains, extra virgin olive oil, and wine characterize the Mediterranean Diet of the early 1960s beyond the plant-based foods diets (rich in fruit, vegetable, nuts, whole grains, fats from natural liquid vegetable oils, adequate intake of *n*-3 PUFAs) that are important to prevent CVD and other diet-related chronic diseases [77]. Among the scores examined in this review, only the DS and MSDPS are coherent with qualitative characteristics of Mediterranean Diet of the early 1960s.

### 5.2. Quantitative Score Divergences from Traditional Mediterranean Diet

In the MDS and some of its variants (Table 2) the gender-specific medians are used as cut-offs to distinguish between levels of intakes of each score component that will be scored 0 or 1. However, the value of the median does not necessarily reflect a level of intake of foods that is consistent with a positive or negative effect on health [67]. For example, it is well established that the intake at least of 3 servings of whole grains replacing refined grains is necessary to have CVD benefits [52,78]. If the intake of whole grains is low in the examined population, the value of 1 point assigned to people with a consumption level at or above the median should be inappropriate in any case, since it does not meet the protective value. In the same way, if there is a large intake of a non-Mediterranean food in the population, assigning a value of 1 point to people whose consumption is below the median should not be considered adequate since the intake could still be excessive. In agreement with the concept expressed by other authors, the median is just a value of position that divides a distribution in 50% below and 50% above the value of the median [67]. Besides, since the median values may be different among populations, the results of one study could not be comparable with those of another study performed in a different population [67]. It is noteworthy that the yes/no approach of this score implies an important loss of information. Indeed, the use of a large number of cut-off points (at least more than two) is recommended to improve the diagnostic capacity of a score [79] since the diagnostic accuracy of an index increases as the number of partitions of its components increases [80]. The DS, the r-MED, the Italian Mediterranean Index, the a priori Mediterranean Dietary Pattern, the MAI, and the MSDPS are consistent with this concept. The diagnostic accuracy of an index increases also when each component of the score is assigned with a specific “weight” that depends on the strength of the relationship that each component has with the binary outcome under study. Each component of a composite score contributes differently to the risk of a specific disease [80]. For example, there is some evidence that vegetables have a lower protective effect on CVD than whole grains or fruit [47]. So, “the development of weighted dietary indexes that adequately assess a dietary pattern and its relationship to the burden of a disease is considered essential” [81]. Some authors propose that the “weights” are the ORs obtained from univariate logistic regression [80], or from multiple logistic regression models with each component of the score entering as an independent variable and total score made by remaining components entered in the model, or by multiplying the “weights” obtained from the ORs with the inverse of the variance of the specific OR, which represents the effect size of the association [82].

## 6. Conclusions

In conclusion, our study shows that the Mediterranean Diet is a useful tool to reduce the risk of CVD as clearly shown by some systematic reviews and meta-analyses [83,84]. In a recent meta-analysis, Sofi *et al*. found that a two-point increase in adherence score for the Mediterranean Diet leads to a 10% reduced risk of CVD [84]. However, published studies show quite different effects from the Mediterranean Diet with regard to specific conditions such as CHD and cerebrovascular disease. We cannot exclude that several factors are responsible for these differences, such as a small number of cases in some studies, different control of confounders, or a different dietary assessment among the studies, and a lack of repeated measurement of a Mediterranean-style diet [85]. In our opinion, the use of *a priori* dietary indexes established to comply with the characteristics of the Mediterranean Diet of the early 1960s could be useful to better understand the effectiveness of this dietary pattern in managing CVD risk. A valid score for studying the relationship between the traditional Mediterranean Diet of the early 1960s and CVD should have the quantitative and qualitative characteristics of the true early dietary pattern as a reference. However, some of the health effects of the traditional Mediterranean Diet might be due to some characteristics which are not easily quantified: some cooking styles that preserve water-soluble nutrients, the consumption of fresh and not greenhouse vegetables which are poorer in phytochemicals, the consumption of fresh fruit at the end of meal which can counteract the pro-inflammatory and pro-oxidant effect of foods [86], the use of spices and herbs that may improve CVD risk factors [87], the use of stone ground sourdough bread whose richness in fiber and low glycemic index make it very healthy [88], and so on.
